# Supplementary material for: User Perceptions and Needs Analysis of a Virtual Coach for Active and Healthy Ageing—An International Qualitative Study
Source: Int J Environ Res Public Health. 2022 Aug 19;19(16):10341. doi: 10.3390/ijerph191610341 (PMC9408500; doi:10.3390/ijerph191610341)
Supplement: Supplementary file 1 [file ijerph-19-10341-s001.zip › ijerph-1835559-supplementary.pdf]

Supplementary Materials

Table S1. The individual main categories and subcategories broken down by country.

	Country	Institution	Recruitment through	Interviewer characteristivs	Interview mode	Obtaining informed consent
Europe	Germany	Diocesan Caritas Association for the Archdiocese of Cologne (DiCV)	The participants were recruited in April 2021, on the one hand, via the e-mail distribution list of the open help for the elderly of the DiCV and, on the other hand, via the newsletter of the German National Association of Senior Citizens' Organisations (Bundesarbeitsgemeinschaft der Seniorenorganisationen e.V., BAGSO). Both the DiCV and the BAGSO published a call for interview partners with essential information about the e-VITA project (goals, duration, content) and the interview (duration, content, anonymization). In addition, the contact details of the responsible project consultant of the DiCV were given, so that interested potential interview partners could contact her independently.	The interviewer (the Autor J.M) was 27 years old at the time, is female and has a master's degree in health care science as well as experience in conducting qualitative interviews	The interviews were conducted via the Zoom video conferencing platform (Zoom Video Communications, Inc.). All participants were in their homes during this process, but no other people were present. The cameras of both parties were switched on. In parallel, the audio track of the interview was recorded via the dictation mode of a Samsung Galaxy A40 (the interviewer's work mobile phone); the participants were informed that only an audio and no video recording would take place. The transcription of all interviews was carried out by an external service provider and followed the rules of. The transcripts were not sent separately to the interviewees for review, but the participants will be informed about the results of the interview study, as they wish.	The information letter was then sent to the potential participants by email for initial information and additionally by post if respondents were interested in being interviewed. Respondents then returned the signed informed consents using an enclosed return envelope. There were no costs for the respondents.
	France	Assistance Publique –	Participants were recruited in March 2021. An e‐mail was sent by the researchers to	A male doctor in Psychology with	The interviews were conducted via the Skype video	

		Hôpitaux de Paris (APHP)	the participants of the workshop “multimedia Coffee” of the Broca Living Lab – APHP. This workshop “multimedia Coffee” familiarize seniors with the information and communication technologies for daily use; offer a space for listening and exchanging information on age - related disorders and technological aids; allow a meeting between senior, health professionals and creators who will provide the assistive technologies of tomorrow. In the mail sent to participants, essential information about the project e - VITA was indicated (goals, duration, content), as well as the information about the interview (duration, content, anonymization). Persons interested in this project were invited to contact the researchers by e - mail or by phone call	experience in conducting qualitative interviews	conferencing platform. During the interviews the participants were in their own homes, in a quiet room. The cameras of both protagonists were switched on. The sessions were recorded using the video recording tool of the Skype platform. The participants were informed about the audio and video recording of the interview. Due to technical problems, one interview was conducted and recorded using an Honor mobile phone (the interviewer's personal phone); another took place at the Broca Living Lab premises and was also recorded using the same mobile phone.	
	Italy	Istituto Nazionale di Riposo e Cura Anziani (INRCA)	In March 2021, the psychologist contacted primary end - users of the network of INRCA, asked them some screening questions for checking their inclusion characteristics, explained the project and the interview (duration, content, anonymisation) and proposed them to take part in it. Then, an appointment to conduct the full questions was planned.	A trained female psychologist was responsible of conducting the interviews. She has experience both in dealing with older adults an in conducting qualitative research.	Due to the Covid19 restriction, a mix between physical and online meeting was managed: three interviews were conducted face - to - face, the other five via telephone. All interviews were recorded.	The eight individuals who accepted to participate were provided with an information letter and informed consent to data treatment. The psychologists clarified that participants had to sign two copies of the informed consent since one

						copy and the information letter remained in the possession of the participant
	Tohoku, Sendai	Tohoku University	Participants for in-person interviews were recruited from existing contacts who had expressed their desire to be available for future experiments at the university, and from paper advertisements placed at local city district ward offices. Zoom participants were recruited from an advertisement placed in a free newspaper (Kahoku Shinpo – a daily newspaper published in Sendai)	interviews were conducted in rotation between two interviewers. Both interviewers were female, aged 30 and 55 respectively. Neither had experience in conducting qualitative interviews, although one had experience as an experiment tester	A total of 12 interviews were conducted in-person at the Institute of Development, Aging and Cancer, Tohoku University, Sendai. An additional 5 interviews were conducted via the Zoom video conferencing platform (Zoom Video Communications, Inc.). All participants were in their homes during this process, and no other people were present. In all cases, the cameras of both interview participants were switched on. In parallel, the audio track of the interview was recorded via the voice memo application of an iPhone 8 / iPhone 6 (the interviewer's work mobile phones); the participants were informed that only an audio and no video recording would take place.	
Japan	Aichi	National Center for Advanced and Specialized		the interviewer was a female occupational therapist who has worked at a hospital for 15	The camera was turned on for both parties during the online interview, but only the audio was recorded using an IC-Recorder, the same as for the in - person interviews.	

		Medicine (NCGG)		years. She learned how to do medical interviews, and routinely conducts interviews with community - dwelling older people and patients of all age groups (i.e., from children to older people) as a professional	Participants were informed about this.	
	Tokyo	Institute of Gerontology, J. F. Oberlin University (IGOU)	Interviewees were selected by snow-ball sampling	The interviews with the older subjects were conducted by two interviewers with doctoral degrees in gerontology	Four were conducted face-to-face and four online using Zoom. The interviews were conducted between April and May of 2021. There were no drop-outs and none declined an interview. All interviews were transcribed using the IBM Watson speech-to-text service, and the transcription files were further checked by the interviewers.	Eight participants were provided written and verbal informed consent.
